# Promoting Induced Pluripotent Stem Cell-driven Biomineralization and Periodontal Regeneration in Rats with Maxillary-Molar Defects using Injectable BMP-6 Hydrogel

**DOI:** 10.1038/s41598-017-18415-6

**Published:** 2018-01-08

**Authors:** Ke-Hung Chien, Yuh-Lih Chang, Mong-Lien Wang, Jen-Hua Chuang, Ya-Chi Yang, Ming-Cheng Tai, Chien-Ying Wang, Yung-Yang Liu, Hsin-Yang Li, Jiang-Torng Chen, Shou-Yen Kao, Hen-Li Chen, Wen-Liang Lo

**Affiliations:** 1 Department of Ophthalmology, Tri-Service General Hospital and National Defense Medical Center, Taipei, 114 Taiwan; 2 Institute of Pharmacology, National Yang-Ming University, Taipei, 112 Taiwan; 3 School of Medicine, National Yang-Ming University, Taipei, 112 Taiwan; 4 Department of Pharmacology, Taipei Veterans General Hospital, Taipei, 112 Taiwan; 5 Department of Medical Research, Taipei Veterans General Hospital, Taipei, 112 Taiwan; 6 Institute of Clinical Medicine, National Yang-Ming University, Taipei, 112 Taiwan; 7 Department of Chest, Taipei Veterans General Hospital, Taipei, 112 Taiwan; 8 Institute of Anatomy and Cell Biology, National Yang-Ming University, Taipei, 112 Taiwan; 9 Institute of Oral Biology, National Yang-Ming University, Taipei, 112 Taiwan; 10 Department of Stomatology, Taipei Veterans General Hospital, Taipei, 112 Taiwan; 11 Division of Oral and Maxillofacial Surgery, Department of Stomatology, Taipei Veterans General Hospital, Taipei, 112 Taiwan; 12 Department of Dentistry, School of Dentistry, National Yang-Ming University, Taipei, 112 Taiwan

## Abstract

Periodontal disease may cause considerable destruction of alveolar bone, periodontal ligaments (PDLs) and cementum and even lead to progressive oral dysfunction. Periodontal tissue regeneration is the ultimate goal of periodontal disease treatment to reconstruct both structures and functions. However, the regenerative efficiency is low, possibly due to the lack of a proper periodontal microenvironment. In this study, we applied an injectable and thermosensitive chitosan/gelatin/glycerol phosphate hydrogel to provide a 3D environment for transplanted stem cells and to enhance stem cell delivery and engraftment. The iPSCs-BMP-6-hydrogel complex promoted osteogenesis and the differentiation of new connective tissue and PDL formation. In animal models of maxillary-molar defects, the iPSCs-BMP-6-hydrogel-treated group showed significant mineralization with increased bone volume, trabecular number and trabecular thickness. Synergistic effects of iPSCs and BMP-6 increased both bone and cementum formation. IPSCs-BMP-6-hydrogel-treated animals showed new bone synthesis (increased ALP- and TRAP-positive cells), new PDL regeneration (shown through Masson’s trichrome staining and a qualification assay), and reduced levels of inflammatory cytokines. These findings suggest that hydrogel-encapsulated iPSCs combined with BMP-6 provide a new strategy to enhance periodontal regeneration. This combination not only promoted stem cell-derived graft engraftment but also minimized the progress of inflammation, which resulted in highly possible periodontal regeneration.

## Introduction

Periodontal disease causes considerable destruction of alveolar bone, periodontal ligament (PDL) and cementum as well as excess bone resorption in later stages, which often leads to tooth loss^1^. Periodontal tissue regeneration is the ultimate periodontal disease treatment because it may reconstruct the form and function of the lost tissues. PDL fibers were found to promote periodontal complex regeneration when left unretracted in beagles^2^. Ideally, the regenerated PDL fibers should be inserted into the new cementum to connect the root surface and new alveolar bone. PDL stem cells proved to be ideal tissue sources for periodontal regeneration with the advantage of having differentiation potential to form adipocytes, collagen-forming cells, osteoblast-like cells and cementoblast-like cells. Human PDL stem cells implanted in immunocompromised mice successfully generated cementum/PDL-like structures to promote periodontal tissue repair^3^. However, the acquisition of periodontal stem cells is restricted. Induced pluripotent stem cells (iPSCs) are a powerful regenerative platform to produce patient-specific multi-lineage functional cells and tissues without the concerns of immune rejection when the cells are transplanted. Recent studies showed that iPSCs-derived mesenchymal stem cells may facilitate the repair of periodontal defects by increasing regeneration and the production of newly formed mineralized tissues^4,5^. Nevertheless, the *in vivo* regeneration capability of iPSCs to directly differentiate into periodontal tissue or PDL when implanted in defect sites has yet to be determined.

Bone morphogenetic proteins (BMPs) have been shown to accelerate bone formation and promote periodontal regeneration^6^. Recombinant BMPs, such as BMP-2, induce bone formation in humans^7,8^, and *in vitro* experiments demonstrated that BMP-2 enhanced alveolar bone regeneration and remodeling^9,10^. These reports suggested there was therapeutic potential for BMPs for the management of numerous clinical conditions. However, the effects of BMP-6 on inducing cementum formation were limited^9,10^. Nevertheless, BMP-2 was also implicated in causing tooth ankylosis and root resorption^11^, which has delayed the development of BMP-2 applications for periodontal regeneration. Another BMP member, BMP-6, was shown to be superior to BMP-2. Applying synthetic BMP-6 polypeptides in a rat periodontal fenestration defect model enhanced periodontal wound healing and regeneration along with increases in new bone and cementum formation^12^. Additionally, BMP-6 induced osteogenic differentiation more efficiently than BMP-2 *in vitro* when both were overexpressed in mesenchymal stem cells (MSCs)^13^. However, the role of BMP-6 in iPSCs differentiation in periodontal tissues or PDL is still an open question. Although iPSCs cell therapy is one approach for treating periodontal diseases, extremely low retention and survival rates of implanted cells are still major hurdles. Therefore, a plausible approach for treatment would be to couple osteoinductive and chondrogenesis factors, such as BMP-6, with implanted iPSCs using absorbable biomaterials to enhance bone and cementum regeneration in the injured areas.

A 3D culture of stem cells has advantages for the *in vitro* regeneration of functional tissues because it more closely resembles the physiological orientation of the tissue environment. We developed a novel thermosensitive injectable chitosan/gelatin/glycerol phosphate hydrogel to create a 3D environment for stem cells and to enhance the efficiency of cell delivery^14,15^. Recently, we developed a novel injectable hydrogel that could enhance stem cell delivery and engraftment into injured corneas^14^. A mixture of hydrogel and iPSCs repaired a corneal epithelial wound significantly faster than iPSCs alone^14^. This thermosensitive hydrogel, therefore, may be an ideal bio-scaffold to increase iPSCs engraftment and survival^16^. We have developed a novel injectable hydrogel to enhance stem cell delivery and engraftment in injured corneas using the same methods as in our prior study^14^. Moreover, although evidence has shown the therapeutic potential of BMPs and stem cells in periodontal diseases, the success rate may vary according to therapy retention time due to insufficient exposure in the oral environment. The aims of this study focused on constructing a 3D-periodontal therapeutic environment system that combined iPSCs therapy with hydrogel-BMP6 to reprogram the periodontal defects and remodel periodontal regeneration *in vivo*. To this end, we developed a BMP-6 sustaining releasing system with thermosensitive hydrogel that served as a 3D bio-scaffold for iPSCs to be engrafted and induced into a lineage of periodontal tissues. We validated the capabilities and advantages of using iPSCs combined with BMP-6 in periodontal tissue regeneration in this study, and we further demonstrated the effects of the iPSCs-BMP-6-hydrogel combination in 3D culture as a regenerative biomaterial for periodontal functional repair both *in vitro* and *in vivo*.

## Results

### Evaluation of pluripotency from iPSCs

**Figure 1:**
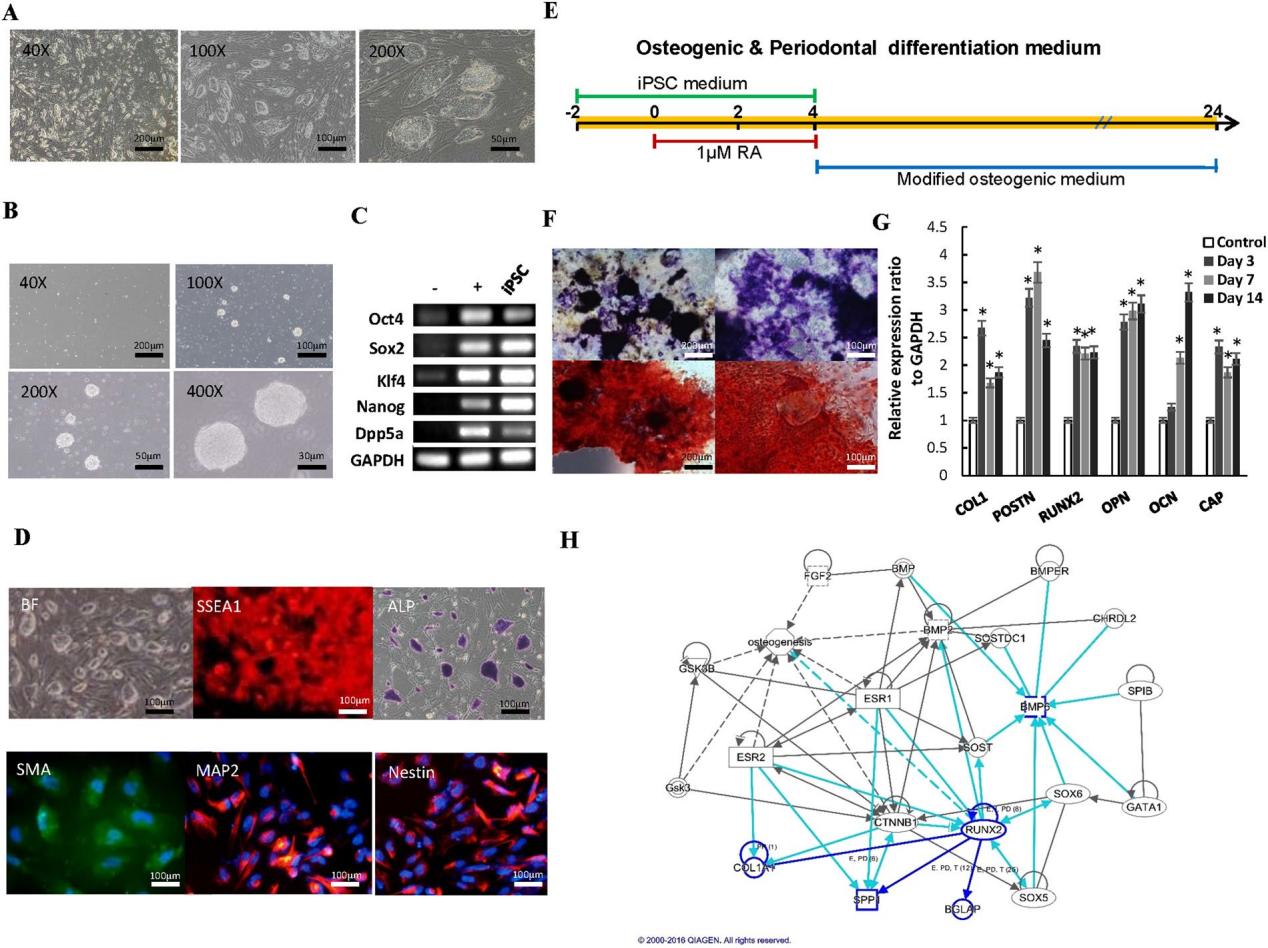
Characterization of fibroblast-reprogrammed iPSCs. (**A**) After transfection with *Oct4/Sox2/Klf4/c-Myc* and culture on REFs as a feeder, the morphology of reprogramming fibroblasts was changed to small, compact colonies. (**B**) After suspension culture, the morphology of EB colonies from iPSCs showed typical round-shaped colony morphology with small, tightly packed cells. (**C**) The RT-PCR showed that colonies of iPSCs expressed *Oct4*, *Sox2*, *Klf4* and *Nanog*. (**D**) Staining of specific markers was positive for SSEA1, ALP, SMA, Map2, Nestin. (**E**) Modified osteogenic induction medium protocol of our study to efficiently induce iPSCs into osteogenic lineages. The unit for the time axis is “day.” (**F**) The osteogenic ability of iPSCs was demonstrated by ALP and ARS staining. (**G**) From RT-PCR, changes in different regeneration-related gene expressions of iPSCs were demonstrated at different time-points. (**H**) The potential regulatory circuits within periodontal regeneration and osteogenesis were generated through the use of IPA (QIAGEN Inc., https://www.qiagenbioinformatics.com/products/ingenuity-pathway-analysis)^18^, of which BMP-6 was documented to drive osteogenesis through the up-regulation of *SOX6* and *RUNX2*, which subsequently promoted the expression of *COL1*, *OPN* (*SPP1*), and *OCN* (*BGLAP*) to eventually trigger the osteogenic process. *p < 0.05.

iPSCs are an alternative source of pluripotent cells that avoid the ethical issues arose in the field of tissue regeneration, and the generation procedure for these cells has been standardized and well established^17^. First, we used fibroblasts from Sprague Dawley rat to generate iPSCs, and these cells progressively formed colonies with increasing sizes during the reprogramming process. These colonies were cultivated on rat embryonic fibroblast (REF) feeder cells in media. After iPSCs were reaching 90% confluency, cell clones were passaged by picking iPSCs colonies with the hanging drop method into non-adherent Petri dishes for EB formation (Fig. 1A and supplementary). After 4 days of suspension culture, a drop-suspension was cultured for 48 h followed by suspension for 3 days. The EB colonies exhibited the typical round shape colony morphology with small, tightly packed cells (Fig. 1B). RT-PCR also showed that iPSCs expressed various stemness genes, such as *Oct4*, *Sox2*, *Klf4*, *Nanog*, and *DPPA5* (Fig. 1C). We then evaluated the pluripotency of iPSCs by measuring EB formation and three germ layer differentiation. Staining of specific markers showed positive signals for stage-specific embryonic antigen-1 (SSEA-1), alkaline phosphate (ALP) in iPSCs, and smooth muscle actin (SMA, mesoderm), neuronal marker (Map2), and Nestin (ectoderm) in differentiated cells (Fig. 1D). We modified prior formulas to induce iPSCs into osteogenic and periodontal differentiation in a more efficient way (Fig. 1E). Osteogenic ability was demonstrated through extracellular ALP and Alizarin red S (ARS) staining in iPSCs after induction in an osteogenic lineage for 14 days. Both results showed strong staining under a microscope (Fig. 1F). The results of quantitative RT-PCR showed that the expression levels of *POSTN* and *OPN* were significantly up-regulated after 7-days treatment with induction medium, compared with non-induction iPSCs (Control) (Fig. 1G). Moreover, we addressed the potential regulatory circuit within periodontal regeneration and osteogenesis which were generated through the use of IPA (QIAGEN Inc., https://www.qiagenbioinformatics.com/products/ingenuity-pathway-analysis)^18^, and found that BMP-6 was closely linked to several markers and effectors involved in bone formation. More specifically, BMP-6 was shown to drive osteogenesis through the up-regulation of *SOX6* and *RUNX2*, which subsequently promoted the expression of *COL1*, *OPN* (*SPP1*), and *OCN (BGLAP*) to eventually trigger the osteogenic process (Fig. 1G). Collectively, these data suggested that iPSCs may be a potential resource to facilitate osteogenic- and periodontal-lineage differentiation, and BMP-6 may be a potential target to modulate periodontal reprogramming.

### Osteogenesis and periodontal tissue gene expression promotion by BMP-6 in iPSCs

**Figure 2:**
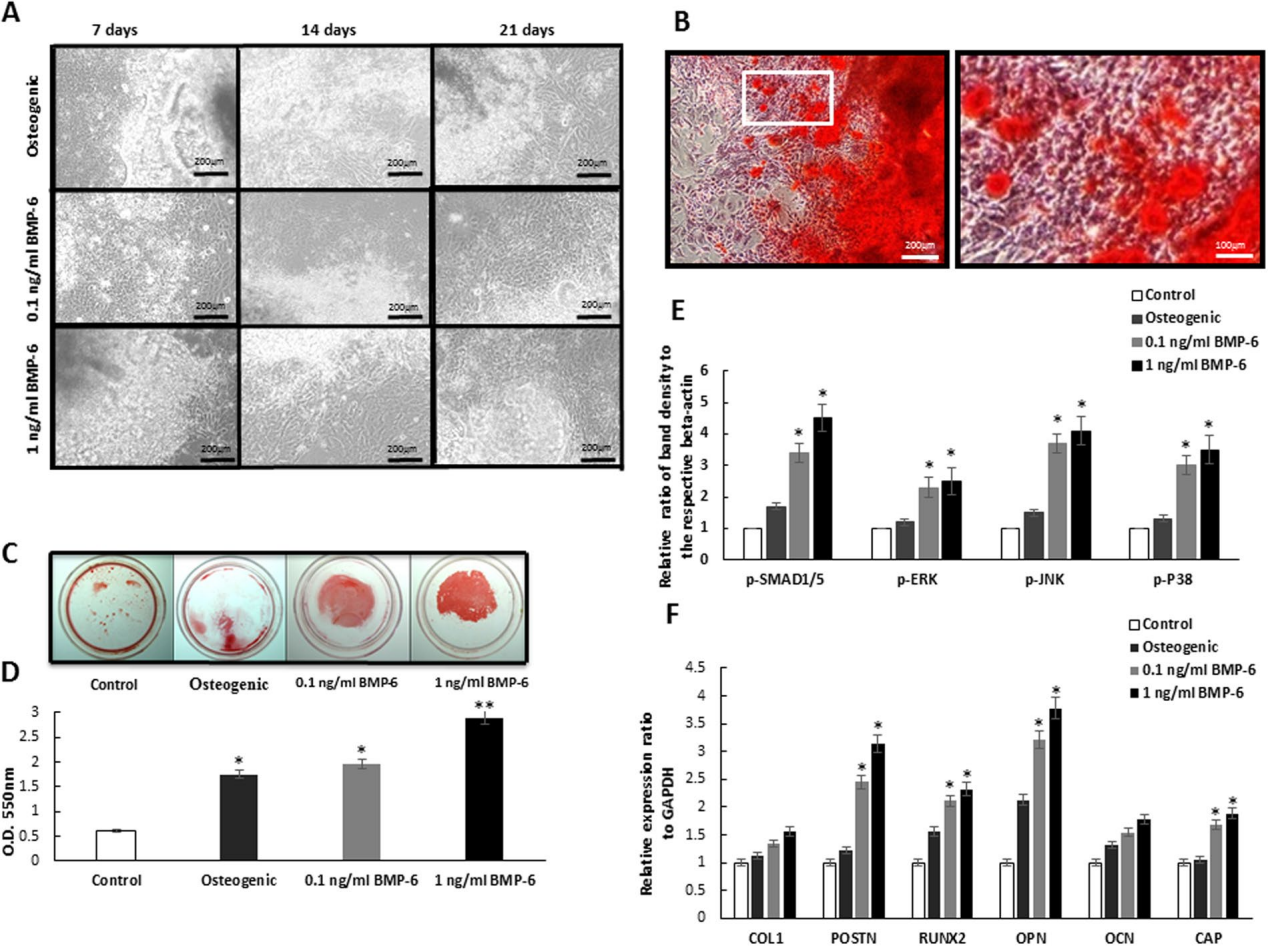
Characterization of BMP-6 induced osteogenesis in iPSCs and its effect on downstream factors. (**A**) Morphology of iPSCs treated with osteogenic medium, 0.1 ng/ml and 1 ng/ml BMP-6 for different time courses (7, 14 and 21 days) observed under a microscope. (**B**) Osteogenic ability was demonstrated by extracellular ARS staining in iPSCs after induction into an osteogenic linage for 28 days. Strong ARS staining was noted under the microscope in 1 mg/ml BMP-6 treated iPSCs. (**C**) Differences in the osteogenic ability of iPSCs treated with different media (control, osteogenic medium, 0.1 ng/ml and 1 ng/ml BMP-6) for 28 days. (**D**) Quantification assay of ARS staining in iPSCs treated with different solutions for 28 days. (**E**) Quantification of west blotting on expression ratios of band density to respective beta-actin of phosphorylated forms of downstream kinases (*p*-SMAD1/5, *p*-ERK, *p*-JNK, and *p*-P38) related to BMP-6 and osteogenesis for 28 days. (**F**) Significantly increased expressions of transcription factors *POSTN*, *RUNX2*, *OPN* and *CAP* in groups of 0.1 ng/ml and 1 ng/ml BMP-6 treated iPSCs or 28 days in quantitative RT-PCR, *p < 0.05.

To evaluate the osteogenesis-induction ability and periodontal effects of BMP-6 in iPSCs, we treated iPSCs with different BMP-6 concentrations for 21 days. Significant osteogenesis differences were noted in 1 ng/ml BMP-6-treated iPSCs compared to cells treated with osteogenesis medium alone and 0.1 ng/ml BMP-6 for 21 days under a microscope (Fig. 2A). The osteogenic ability was then demonstrated by extracellular ARS staining over iPSCs after induction into an osteogenic linage for 28 days. Strong ARS staining was noted under a microscope in 1 ng/ml BMP-6 treated iPSCs (Fig. 2B). Then, we applied ARS staining to compare the different osteogenic ability of iPSCs after adding three different media. With the 10% (w/v) cetylpyridinium chloride (CPC) in 10 mM sodium phosphate for 15 minutes at room temperature, the strongest ARS staining was observed in the 1 ng/ml BMP-6 group, which had best mineralization ability among the groups (Fig. 2C,D), followed by the 0.1 ng/ml BMP-6, osteogenic medium-only and control groups (Fig. 2C,D). We then analyzed downstream factors related to BMP-6 functions and noted significantly higher expression ratios in both 0.1 ng/ml and 1 ng/ml BMP-6 treated iPSCs in phosphorylated forms of downstream kinases (*p*-SMAD1/5, *p*-ERK, *p*-JNK, and *p*-P38) compared to the osteogenic medium-treated group and control group. (Fig. 2E). Next, we evaluated the induction activity of BMP-6 in iPSCs in different lineages. Through RT-PCR, we found not only that osteogenesis-related gene expressions were higher in BMP-6-treated iPSCs (*RUNX2*, *OPN*, *OCN*) but also that the expression of the cementum-related gene, *CAP*, was significantly up-regulated (Fig. 2F). Taken together, these findings show that the *in vitro* administration of BMP-6 effectively up-regulated the periodontal tissue-specific gene expression in a dosage-dependent manner for treating iPSCs, which suggested that BMP-6 played a novel role in promoting periodontal regeneration.

### Preparation of BMP-6-releasing hydrogel and its osteogenesis effects in maxillary-molar defect animal models

**Figure 3:**
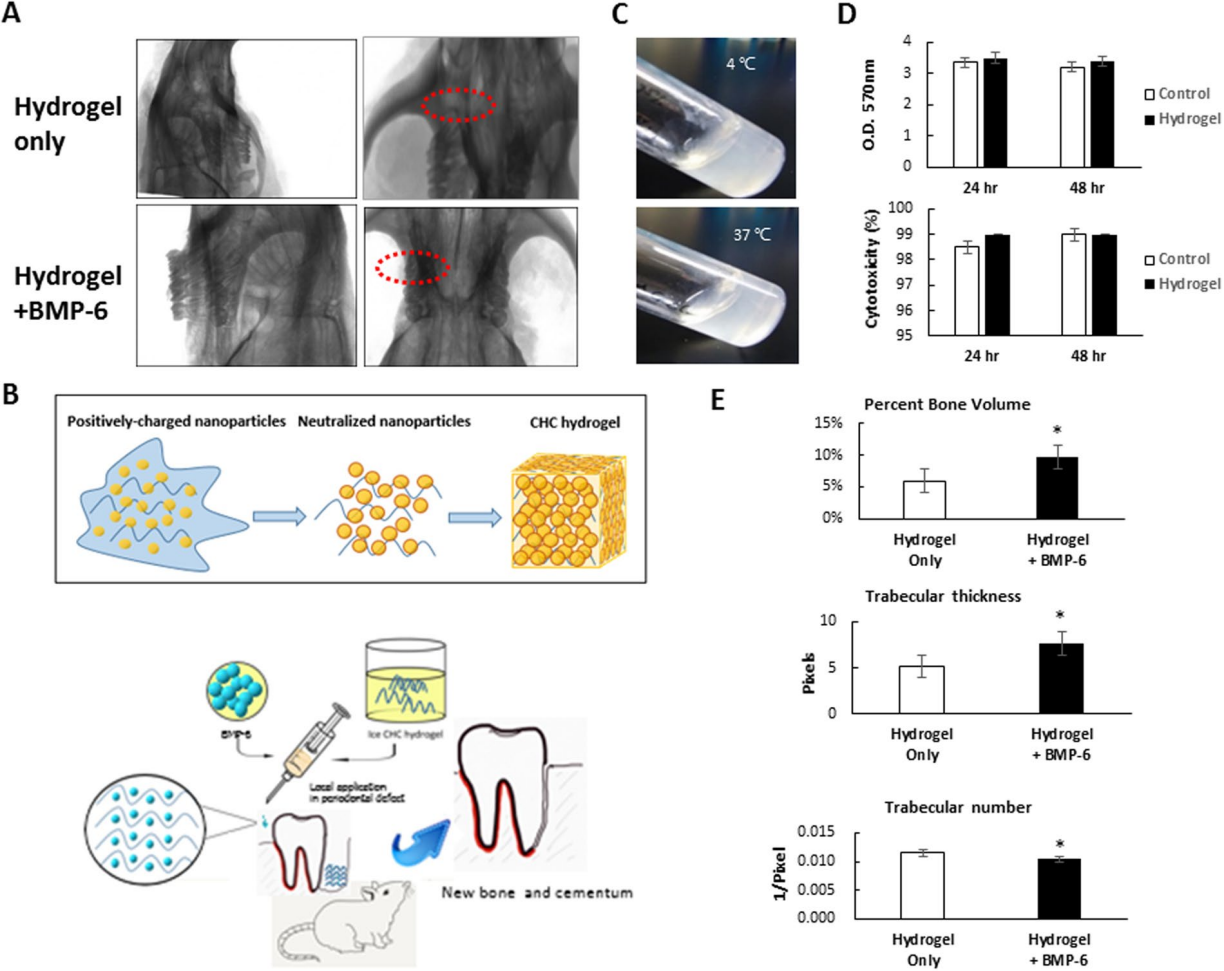
Characterization of BMP-6-releasing hydrogel (BMP-6-hydrogel). (**A**) Different osteogenic effects of the BMP-6-hydrogel group and hydrogel-only group from x-rays. (**B**,**F**) Comparison of the treatment efficacy of hydrogel with and without BMP-6 as a delivery vehicle in rat periodontal defects in parameters of percent bone volume, trabecular thickness, and trabecular number. (**C**) Concept of the thermosensitive hydrogel and mixed with BMP-6 10 ng/ml. (**D**) Transformation of BMP-6-hydrogel from hydrogel status at 4 °C to liquid status at 37 °C. (**E**) Biocompatibility of hydrogel to iPSCs in a crystal violet assay (upper) and lactate dehydrogenase (LDH) assay (lower), *p < 0.05, **p < 0.01.

With the advantage of thermosensitive properties, we then examined whether the hydrogel could be an effective carrier of BMP-6 for sustaining BMP-6 function in periodontal defects. We first prepared a nanoscale hydrogel containing BMP-6 by incorporating BMP-6 peptides into hydrogels (Fig. 3B). The BMP-6-incorporated thermosensitive hydrogel was then applied to periodontal defects in rats. One of the major advantages of the hydrogel was that it could remain gel form at 4 °C and transform into liquid at 37 °C (Fig. 3C). To evaluate the cytotoxicity of the hydrogel, we conducted a crystal violet assay and lactate dehydrogenase (LDH) assay in iPSCs. The results of both the crystal violet and LDH assays showed no significant differences between the control (mESC media only) and experimental groups (hydrogel extraction) (Fig. 3D), which suggested that the hydrogel had little cytotoxicity toward the iPSCs. Next, we evaluated the osteogenic ability of BMP-6 in periodontal defects. We chose 10 ng/ml as an optimal BMP-6 concentration to be incorporated into hydrogel for *in vivo* experiments. After 6 weeks of treatment, osteogenesis identified by x-rays was significantly higher in rats treated with 10 ng/ml hydrogel/BMP-6 than in rats treated only with hydrogel (Fig. 3A). Measurements in osteogenesis within periodontal defects were conducted to determine the percentage of bone volume, trabecular thickness and trabecular number, and they were all significantly different between the hydrogel/BMP-6 and hydrogel-only groups (Fig. 3E). Taken together, these findings showed that BMP-6-conjugated hydrogel presented greater efficiency for promoting osteogenesis than hydrogel alone.

### Preparation of BMP-6-releasing iPSCs-containing hydrogel as an osteogenic scaffold in an animal model of maxillary-molar defects

**Figure 4:**
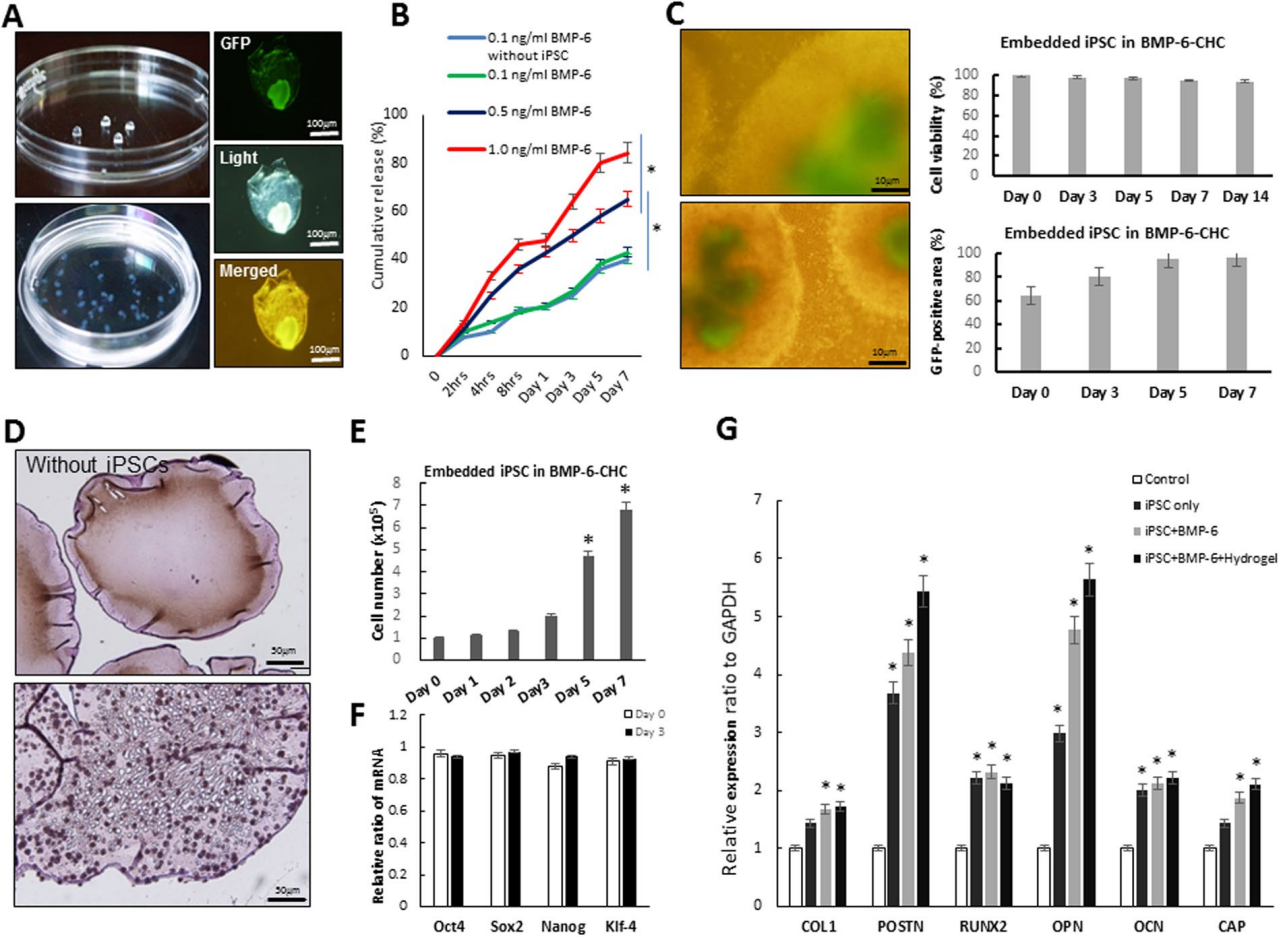
Characterization of BMP-6-releasing iPSCs-containing hydrogel (iPSCs-BMP-6-hydrogel) *in vitro*. (**A**) Morphology of BMP-6-hydrogel-cultivated iPSCs under a microscope. (**B**) The kinetics of BMP-6 release showed that dose-dependent release of BMP-6 at different concentrations at first 7 days. (**C**) Detection of increasing GFP signals revealed regular growth of iPSCs. The MTT assay showed the viability of iPSCs in the presence of BMP-6-hydrogel. (**D**) Morphology of hydrogels that did not contain iPSCs (upper) and hydrogels that contain iPSCs (lower). (**E**) GFP-expressing iPSCs proliferated in the hydrogel in the presence of culture media for 7 days. (**F**) Quantitative RT-PCR results indicated that various stemness genes were expressed in the presence of BMP-6-hydrogel for 3 days. (**G**) Quantitative RT-PCR results indicated significantly higher expressions of *POSTN* and *OPN* genes in the iPSCs-BMP-6-hydrogel group, *p < 0.05.

Because hydrogel can stably deliver BMP-6 while significant osteogenic events were found within treated periodontal defects (Fig. 3), we investigated whether iPSCs could potentiate the induction ability of BMP-6. We created BMP-6-releasing iPSCs-containing hydrogel at a density of 2 × 10^4^ iPSCs cells/20 μl hydrogel and then evaluated the utility of BMP-6-hydrogel as a vehicle for iPSCs delivery^14^. We assessed the fate of iPSCs cultivated in BMP-6-hydrogel in which the iPSCs were co-transfected with a vector encoding pCX-EGFP for expression of green fluorescence protein (GFP)^14^. The GFP-labeled iPSCs exhibited a slightly shrunken morphology (diameter = 1 mm) in the BMP-6-hydrogel compared to iPSCs cultivated in conventional culture media (Fig. 4A,C). Cultivation of iPSCs within the BMP-6-hydrogel did not affect the kinetics of BMP-6 release (Fig. 4B). The release kinetics of BMP-6 from the BMP-6-hydrogel created with different concentrations of BMP-6 were evaluated by measuring the cumulative percentages of released BMP-6 at different time points within seven days. The BMP-6-hydrogel complex was capable of steady secretion of BMP-6 into the culture media in a dose-dependent manner (Fig. 4B). A BMP-6 concentration of 1.0 ng/ml was selected as the optimal concentration in hydrogel for additional *in vitro* experiments. During the cultivation period within the BMP-6-hydrogel treatment, the GFP signal from GFP-labeled iPSCs gradually increased, which indicated the growth of iPSCs in this environment (Fig. 4F). An MTT assay also confirmed the viability of iPSCs (Fig. 4C). Quantitative RT-PCR revealed that several stemness genes, including *Oct4*, *Sox2*, *Nanog* and *klf-4*, were stably expressed and remained unchanged upon BMP-6-hydrogel treatment within at least 3 days (Fig. 4F). Furthermore, significantly up-regulated *POSTN* and *OPN* gene expressions were noted in iPSCs-BMP-6-hydrogel cells compared to either iPSCs-BMP-6, iPSCs only or the control group (Fig. 4G). Collectively, BMP-6-hydrogel was capable of a sustained BMP-6 release that rendered this hydrogel an optimal vehicle for iPSCs to maintain their biology and regular growth.

### Micro-CT (μ-CT) analysis to evaluate the osteogenesis effects of BMP-6-releasing iPSCs-containing hydrogel in an animal model of maxillary-molar defects

**Figure 5:**
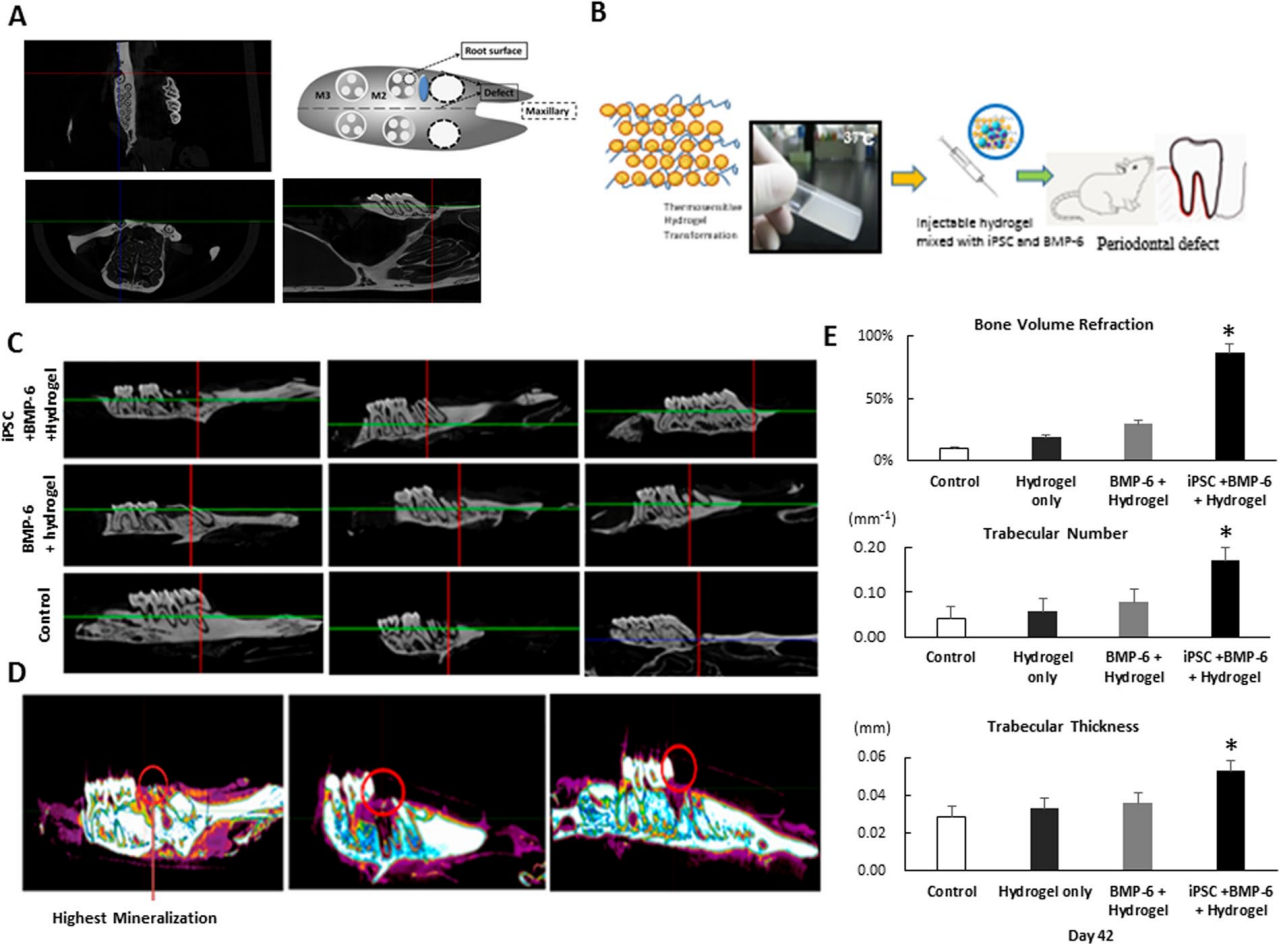
Osteogenic effects of iPSCs-BMP-6-hydrogel *in vivo*. Depiction of rat periodontal defect design. The transverse plane illustrating the defect was created at the mid-root level of M2 and M3. Micro-CT imagining showed different planes for the periodontal defects. (**B**) A combination of hydrogel (gel form), iPSCs and BMP-6 (10 ng/ml) was applied to rat periodontal defects. (**C**) Different new bone formation was demonstrated among different groups (control, BMP-6-hydrogel, iPSCs-BMP-6-hydrogel) from different views of micro-CT images. (**D**) The highest osteogenic ability was noted in the iPSCs-BMP-6-hydrogel treated rats from micro-CT images. (**E**) The highest osteogenic ability was found in the iPSCs-BMP-6-hydrogel-treated group relative to other groups in the parameters of bone volume refraction, trabecular number, and trabecular thickness, *p < 0.05.

Because the iPSCs-BMP-6-hydrogel specimens exhibited up-regulated gene expressions in RT-PCR (Fig. 4), we evaluated the osteogenic effects of this treatment *in vivo*. Periodontal defects were created over rat maxilla as previously described (Fig. 5A)^19^, and hydrogel was mixed with iPSCs and 10 ng/ml BMP-6 for post-operative injection into periodontal defects (Fig. 5B). The 3D restructured and sectioned images were collected by micro-CT at 6 weeks post-operation to investigate the osteogenic inductivity under different experimental settings, such as iPSCs-BMP-6-hydrogel, BMP-6-hydrogel, hydrogel only and the control group (Fig. 5C).

**Figure 6:**
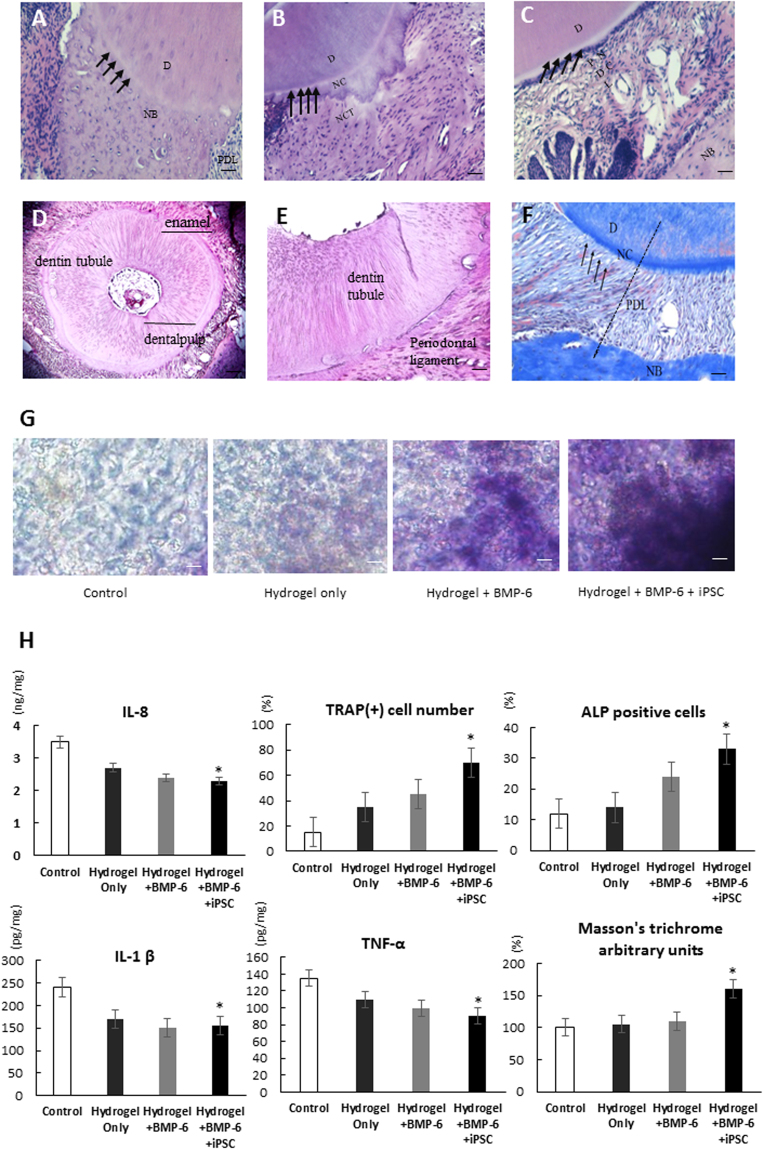
Histology and immunohistochemistry of iPSCs-BMP-6-hydrogel effects *in vivo*. (**A**–**C**) Specimens from the center of the defect under 200x magnification using hematoxylin and eosin staining. (**A**) The BMP-6-only group showed new bone (NB) formation, (**B**) the BMP-6 with hydrogel (BMP-6-hydrogel) group showed both new connective tissue (NCT) and NB formation, and (**C**) the iPSCs with BMP-6 and hydrogel group (iPSCs-BMP-6-hydrogel) showed not only new cementum (NC, arrow head) formation but also new periodontal ligament (PDL) formation. (**D**,**E**) Transverse sections of regenerated tissues, low- and high-power field views in the iPSCs-BMP-6-hydrogel group. (**F**) The iPSCs-BMP-6-hydrogel group visualized with Masson’s trichrome staining to determine the collagen components of regenerated tissues (left to dot line, right to dot line is un-operated area for comparison). (**G**) ALP staining of each group demonstrated increased ALP-positive cells in the iPSCs-BMP-6-hydrogel group. (**H**) Decreased fractions of inflammatory cells IL-8, TNF-α and IL-1β and increased fractions of ALP-positive cells and TRAP-positive cells in the iPSCs-BMP-6-hydrogel group compared to other groups. Only the iPSCs-BMP-6-hydrogel group showed significantly stronger Masson’s trichrome staining. D, dentin; NC, new cementum; NB, new bone; PDL, periodontal ligament; NCT, new connective tissue; Scale bar = 100 μm, *p < 0.05.

From the section views, more detailed information about new bone formation was provided (Fig. 5C), and significantly more regenerated bony tissues were identified in the iPSCs-BMP-6-hydrogel-treated group (Fig. 5D). In qualitative measurements after 6 weeks post-transplantation, only the iPSCs-combined BMP-6 group and hydrogel (iPSCs-BMP-6-hydrogel) showed significantly greater bone volume refraction relative to other groups (Fig. 6E). With respect to trabecular numbers, the iPSCs-BMP-6-hydrogel group provided significantly higher trabecular numbers (Fig. 6E), and similar results were observed for trabecular thickness. Within six weeks, the iPSCs-BMP-6-hydrogel group demonstrated a rapid bone bridging with a large volume of regenerated bone tissue within periodontal defects. Collectively, iPSCs combined with the BMP-6 and hydrogel group (iPSCs-BMP-6-hydrogel) showed the highest osteogenic abilities in different measurements through micro-CT analysis six weeks post-implantation.

### Histomorphometric analysis of BMP-6-releasing iPSCs-containing hydrogel in animal models of maxillary-molar defects

Because significant osteogenic ability was noted within the iPSCs-BMP-6-hydrogel group (Fig. 5), we examined the induction of the soft tissue regeneration ability of this formation *in vivo*. H & E staining revealed that the BMP-6 treated group (10 ng/ml) showed new bone (NB) formation around cementum (Fig. 6A). Additionally, BMP-6 with the hydrogel group (BMP-6-hydrogel) showed both new connective tissue (NCT) formation and NB formation (Fig. 6B). Moreover, iPSCs combined with BMP-6 and hydrogel (iPSCs-BMP-6-hydrogel) showed not only new cementum (NC) formation but also new periodontal ligament (PDL) formation (Fig. 6C). Collectively, NB and NCT formation were found in both the BMP-6-only group and BMP-6-hydrogel group, but only the iPSCs-BMP-6-hydrogel group could induce NB and new PDL formation (Fig. 6D,E,F). As a result, iPSCs demonstrated an add-on advantage to promote new PDL formation in the animal model of maxillary-molar defects, whereas BMP-6 could only induce osteogenesis.

**Figure 7:**
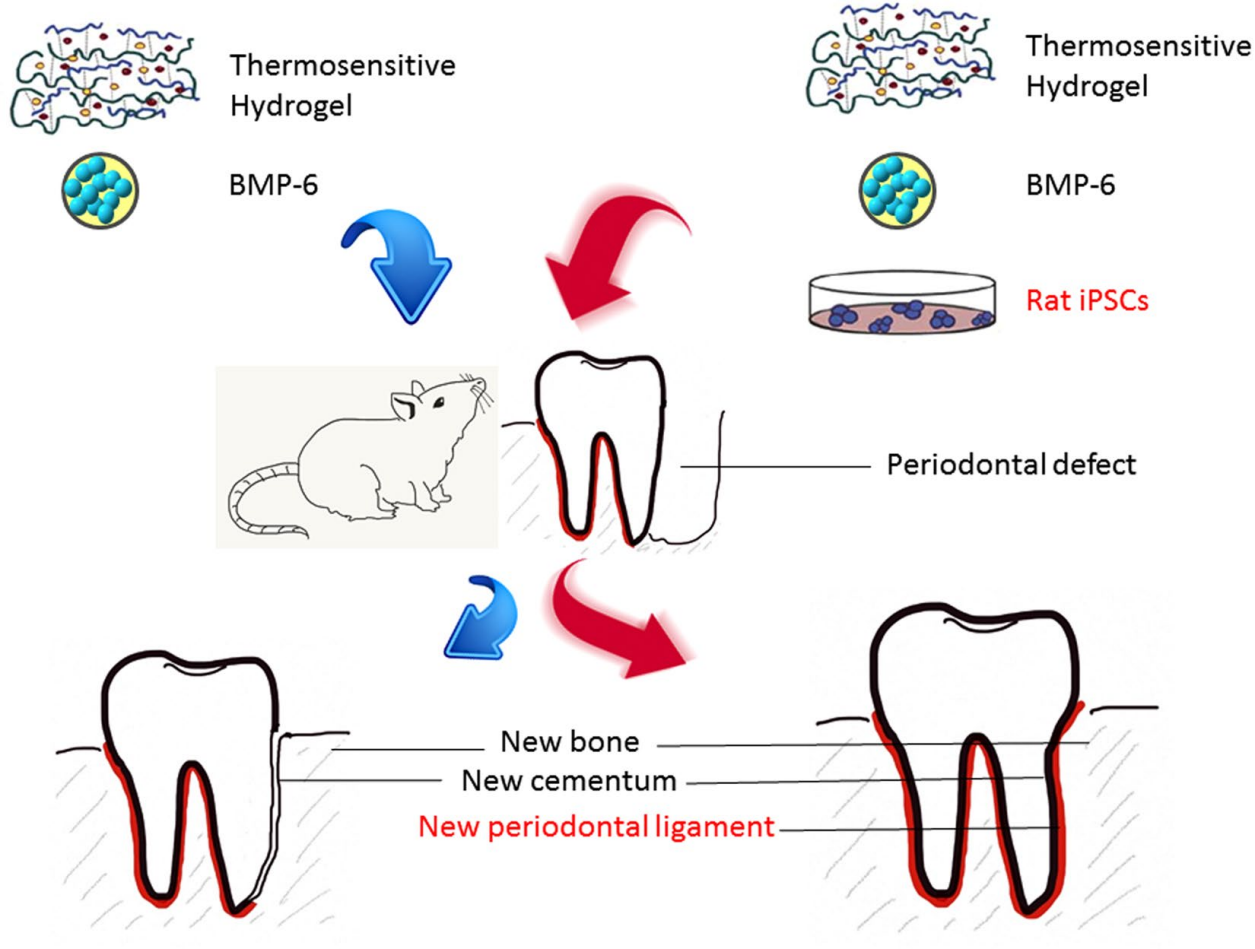
Schematic of the study design. A combination of iPSCs, BMP-6 (10 ng/ml) and hydrogel was designed to be injected into periodontal defects of rats to regenerate layers of cementum (bone and connective tissues) and periodontal ligaments.

Inflammation after graft transplantation is a major problem that leads to graft failure. Both inflammatory cells and inflammation-related cytokines, including TNF-α, IL-1β and IL-6, caused tissue edema and resulted in graft failure. Grafts with iPSCs were shown to not only reduce tissue edema by directly inhibiting inflammatory cells but also effectively regulate the production of inflammatory cytokines in animal models of stroke^20^. To further validate whether iPSCs were capable of suppressing the inflammatory process and remodel the periodontal environment, we injected the iPSCs-BMP-6-hydrogel in animal models of maxillary-molar defects and analyzed the effectiveness of this approach with cytokine arrays and a histo-anatomical survey. We found that inflammatory cytokines (IL-8, TNF-α and IL-1 β) were down-regulated in the iPSCs-BMP-6-hydrogel group compared to hydrogel-only and BMP-6-hydrogel groups. Moreover, the results of histopathological staining showed that the markers for osteoblasts (ALP staining) (Fig. 6G), osteoclasts (TRAP staining) and PDL formation (Masson’s trichrome staining) were all significantly up-regulated in the iPSCs-BMP-6-hydrogel group compared to the other two groups (Fig. 6H). Collectively, these findings showed that iPSCs-BMP-6-hydrogel formation could not only induce osteogenesis but also soft tissue regeneration, including cementum and PDL. These data indicated that iPSCs-BMP-6-hydrogel not only could promote stem cell-derived graft engraftment but also minimize the progress of inflammation, resulting in stem cell reprogramming and periodontal regeneration *in vivo*. To summarize our concept, we illustrated our methodology for using a hydrogel-iPSCs-BMP6 3D bio-scaffold to treat periodontal disease in a regenerative fashion as a schematic illustration shown in Fig. 7.

## Discussion

Three-dimensional (3D) culturing has become a novel strategy to generate tissues like oral mucosa, osteoclasts, and cardiomyocytes for regenerative therapy^21–23^. Compared to single-layer 2D culturing, 3D structures allow cells to freely distribute in a manner similar to the natural microenvironment. Evidence has supported the concept of using 3D cultures for cell therapeutic strategies, and most of these studies used the extracellular matrix or its analogues as 3D culture scaffold^24,25^. Furthermore, the induction of iPSCs differentiation into specific lineages may be of importance not only for applying the cell therapy clinically but also to decrease the risk of pathogenic issues of iPSCs-associated cell therapy. Therefore, 3D culture of iPSCs is needed in regeneration medicine to reconstruct lost tissue without teratoma formation. Periodontal disease results in the destruction of alveolar bone, periodontal ligament (PDL) and cementum and can even lead to tooth loss. However, current therapies can only replace lost teeth or control local periodontal disease. Our data demonstrated that BMP-6 could induce osteogenesis to reconstruct bony tissue and that iPSCs could further promote stem cell engraftment by both minimizing inflammatory progress in the microenvironment and expanding the regenerative lineage to fully reconstruct lost tissues. Collectively, our study demonstrated highly possible periodontal regeneration with an iPSCs-BMP-6-hydrogel combination in an animal model of maxillary-molar defects both *in vitro* and *in vivo*.

Induced pluripotent stem cells (iPSCs) possess pluripotent potential that can be differentiated into any type of organ or tissues and applied as a biomedicine strategy for curing diseases and tissue repair. However, there is a concern that iPSCs may induce teratoma formation and have a tumorigenic potential^26^. Therefore, many recent studies have tried to modify iPSCs induction processes to reduce the risk of teratoma formation^27^. A recent report suggested that cartilage defects from human iPSCs can be repaired using a scaffold-less model employing the subcutaneous transplantation of hiPSCs-derived particles^17^; tumors or ectopic tissues were not found in the recipient mice. Here instead of the generation of specific tissue types or cells, we implanted osteogenic BMP-6 and iPSCs, which only differentiated into mesenchymal lineages of bone, in surgically created periodontal defects. No tumors or ectopic tissues were found in our study objects during the experimental period. Our results demonstrated that iPSCs could be applied as a biological material to provide “*in situ* multilinear differentiation.” Previous studies applied a synthetic BMP-6 peptide to a periodontal fenestration defect in rats that enhanced periodontal regeneration. In this study, we applied human recombinant BMP-6 combined with iPSCs to rat periodontal defects and we examined the synergistic effects of iPSCs and BMP-6 on periodontal regeneration. Despite several studies suggested that BMPs may modulate osteogenesis and cementogenesis in periodontal wounds, native BMPs delivered in a collagenous matrix has been reported to induce cementum, periodontal ligament and alveolar bone regeneration^6^. BMP-6 was a more potent stimulator of bone formation than BMP-2 and -4 in rat osteoblasts^28^. These results indicated that BMP-6 exerted an osteoinductive effect at least in part through the IGF-1 and EGF pathways^29^, which was observed both in a murine model of osteopenia and in the human osteoblastic cell line as well as through the application of BMP-6/ACS onto critical-size supra-alveolar defects that enhanced periodontal wound healing/regeneration. The constructed BMP-6 at low concentration as 0.25 mg/ml significantly supported cementum and bone formation and establishment of a functionally oriented periodontal ligament^30^. BMPs have recently noted to involve in MAPK/ERK/p38/JNK signaling pathway which is reported to regulate osteoblast differentiation and osteogenesis^30–32^. In our study, 0.1 ng/ml and 1 ng/ml BMP-6 were added to osteogenic media. As a result, we found that BMP-6 induced a dose-dependently enhanced mineralization, with 1 ng/ml BMP-6 exhibiting better mineral formation ability (Fig. 2D). In addition, BMP-6 downstream kinases including p-SMAD1/5, p-ERK, p-JNK, and p-P38 were significantly expressed in the iPSCs groups treated with 0.1 ng/ml and 1 ng/ml BMP-6 for 28 days. The findings suggested that BMP-6 could involve in osteogenesis process (Fig. 2E).

Thermosensitive hydrogels have been shown to be a promising biomedical material for drug release, cell encapsulation, and tissue engineering^31,32^. These hydrogels, when combined with drugs or proteins, may provide sustained systemic or local therapeutic effects and be applied as *in vivo* scaffolds for cell delivery^33–35^. When hydrogels are applied as an implant, they have many advantages over conventional implants, such as minimal invasiveness, easy handling, reduced pain, fewer complications, reduced surgery time and healing period, minimal scarring, and the capacity to conform to irregular defects^31,36^. We developed a novel injectable hydrogel that enhanced stem cell delivery and engraftment into an injured cornea in our prior study^14^. A peptide-modified hydrogel containing iPSCs-derived cardiomyocytes was then found to improve cardiac structure and function in rat models of infarcted hearts^37^. Then, we applied the BMP-6-releasing hydrogel to promote iPSCs-Hep growth and maintenance in damaged liver tissues^38^. Recently, a VEGF-containing hydrogel with iPSCs-derived endothelial cells was successfully applied in a mouse hindlimb ischemia model to reduce inflammation and promote muscle tissue regeneration^39^. In this study, we used a hydrogel containing iPSCs and BMP-6 based upon the improved stem cell delivery and tissue engraftment as well as the ability of these hydrogels to be shaped easily to fill the periodontal defects. The results for the hydrogel with iPSCs and BMP-6 group supported our hypothesis and demonstrated the best regeneration ability among the test groups.

Considering that host and environmental factors can influence transplanted stem cells to differentiate into various connective tissue cells^40^, evidence suggests that the microenvironment and the surrounding tissue provide nutrients, growth factors, and extracellular matrices to support MCS differentiation^41^. IPSCs combined with enamel matrix derivatives (EMD) groups had better results than EMD-only groups, which suggested that iPSCs and their surroundings might play an important role in periodontal tissue differentiation^42^. Recently, the osteogenic activity of EMD was shown to be mediated by BMP molecules. BMPs have been evaluated to accelerate bone formation and can promote periodontal regeneration^6^. Although cell therapy using iPSCs is one of the options for treating periodontal diseases, the short retention and survival rates of implanted cells are still a major setback. Therefore, the use of biocompatible and absorbable materials coupled with osteogenic factors, such as BMPs, was developed to secure implanted cells to designated regions as well as to promote regenerative functions. In this study, we developed an injectable thermosensitive hydrogel with consistent release of BMP6 and applied as a vehicle for iPSCs delivery. Rather than pre-treatment of iPSCs for local implantation, as previously described^5^, our findings demonstrated that the functional iPSCs using a BMP-6-containing hydrogel as a delivery vehicle may have potential benefits for the management of cementum and periodontal disease. Additionally, the micro-CT results showed that iPSCs combined with BMP-6 showed the greatest increase in bone volume fraction, trabecular thickness and trabecular number compared to the other groups (Fig. 5E). The histomorphometric analysis showed that both of the BMP-6-containing groups could lead to ankylosis, whereas the hydrogel-only group could lead to irregular connective tissue formation around the surgery region. Additionally, the iPSCs combined with BMP-6 could promote new cementum and new PDL formation (Fig. 6A–F). It seemed that when stem cells were transplanted into different locations directly, they might undergo reprogramming of their gene expression and cross-lineage boundaries. The results suggest that the interaction between stem cells and their new microenvironment may be the reasons why iPSCs can promote periodontal regeneration.

Taken together, the findings in this study revealed that the iPSCs + BMP-6 group could promote minimization compared to the BMP-6 group. The iPSCs + BMP-6 group demonstrated new bone formation by using micro-CT imaging. Histomorphological results demonstrated the new periodontal tissue formation. Therefore, we suggest that iPSCs combined with BMP-6 may provide a new strategy for periodontal regeneration.

## Materials and Methods

### Differentiation of iPSCs into osteocyte-like cells

The iPSCs were reprogrammed from Sprague Dawley rat fibroblasts by transduction of retroviral vectors encoding four transcription factors (Oct-4/Sox2/Klf4/c-Myc; OSKM; Supplementary Information)^43^. In brief, undifferentiated iPSCs were routinely cultured and expanded on mitotically inactivated REFs (50,000 cells/cm^2^) in 6-well culture plates (BD, Franklin Lakes, New Jersey, United States) in the presence of 0.3% leukemia inhibitory factor in an iPSCs medium that consisted of DMEM (Sigma-Aldrich, St. Louis, Missouri, United States) supplemented with 15% FBS (Invitrogen, Waltham, Massachusetts, United States), 100 mM MEM nonessential amino acids (Sigma-Aldrich, St. Louis, Missouri, United States), 0.55 mM 2-mercaptoethanol (Gibco, Waltham, Massachusetts, United States), and antibiotics (Invitrogen, Waltham, Massachusetts, United States). For osteogenic induction, iPSCs were cultured in DMEM-LG (Invitrogen, Waltham, Massachusetts, United States) supplemented with 15% FBS, 50 μg/m L ascorbate-2-phosphate, 10 nmol/L dexamethasone, and 10 mmol/L β-glycerophosphate (Sigma, St. Louis, MO) for 2 weeks^44,45^.

### Evaluation of osteogenic ability in differentiated iPSCs

After exposure to conditioned medium or co-culture for periods of 2 weeks, the cells were washed with PBS and fixed with 10% formalin, then washed again three times with PBS. The PBS was removed and freshly prepared ALP (Sigma-Aldrich, St. Louis, Missouri, United States) substrate solution was added to each well. The cells were incubated with the substrate at room temperature for 15 to 20 minutes, after which the reaction was stopped by aspirating the staining solution and rinsing the wells with PBS. The culture medium was removed from each well, and the cells were gently washed 3 times with PBS. The cells were fixed in 4% formaldehyde (Sigma-Aldrich, St. Louis, Missouri, United States) for 15 minutes at room temperature. Then, the fixative was removed and the cells were washed 3 times with diH2O. DiH2O was then removed completely and 1 ml of 40 mM ARS (Sigma-Aldrich, St. Louis, Missouri, United States) was added to each well. The samples were incubated at room temperature for 20–30 minutes with gentle shaking. The dye was removed, and the cells were washed 5 times with diH2O.

### Qualification of mineralization with Alizarin red S staining

iPSCs-derived embryonic bodies (EBs) were cultured for 21 days in three different media, including osteogenic media as well as BMP-6 in two different concentrations and without dexamethasone. After 28 days of culture in three different media, Alizarin red S staining was performed to analyze the newly formed nodules. Briefly, the medium was removed from EBs and the cells were washed three times with PBS. They were then evaluated for calcium production by staining with 40 mM Alizarin red solution. After fixation and staining, the cells were washed three times with PBS. To quantify the staining, 1 ml of 10% cetylpyridinium chloride (Sigma-Aldrich, St. Louis, Missouri, United States) was added to each well and incubated for 20 minutes to elute the stain. Then, 100 μl of this eluted stain was added to 96-well plates and read at 550 nm using a spectrophotometer^46^.

### Preparation of hydrogel thermo-gelling solutions

The preparation of thermosensitive chitosan/gelatin/glycerol phosphate (C/G/GP) was followed as previous protocol^15^. In briefly, the solution 2.5% chitosan (degree of deacetylation >95%, molecular weights j 340,000, Kiotek, Taiwan) with 1% gelatin (G1890, Sigma, USA) were dissolved in 0.1 M acetic acid (242853, Sigma, USA), and 44.4% glycerol 2-phosphate disodium salt hydrate (b-GP, G6251, Sigma, USA) solution was filtered by 0.22 mm filter (Millex-GV, Millipore, USA) for sterilization and further added into the chitosan/gelatin solution.

### Cytotoxicity of thermosensitive hydrogel on iPSCs

The cytotoxicity of G/C/GP hydrogel on iPSCs was examined using an extraction method^15,16^. A total of 0.1 g G/C/GP hydrogel was immersed in 1 ml mESCM in a 48-well culture plate at 37 °C. The supernatant from each well was collected on day 2 for the cytotoxicity test. iPSCs were seeded in 96-well cell culture plates at a density of 5000 cells per well and cultured in mESCM for 48 h. Cells were then cultured in the extraction medium obtained from the developed hydrogel. Crystal violet (HT90132 - crystal violet solution, Sigma-Aldrich) and lactate dehydrogenase (LDH, cytotox96 non-radioactive cytotoxicity assay, Promega, USA) were used to evaluate the cell viability and cytotoxicity of the developed hydrogel on iPSCs at 24 hours and 48 hours. The OD value of crystal violet and the LDH assay were measured at 570 and 490 nm, respectively, with an ELISA reader.

### Animal model

All procedures involving animals were performed in accordance with the 3Rs protocol and according to the institutional animal welfare guidelines of Taipei Veterans General Hospital (Approval number of Institutional Animal Care and Use Committee (IACUC): 2017-019). Sixteen male Sprague Dawley rats (BioLasco, TW) that were 12 weeks old and weighed 450–550 g were selected into four groups (control group, hydrogel-only group, BMP-6 with hydrogel group and iPSCs with BMP-6 and hydrogel group) and treated with pre-anesthetic medication (atropine sulfate, Sintong) before being placed under general anesthesia (intraperitoneal injection of Zoletil 50, Virbac, France). A surgically created periodontal defect was made on the root surface of the maxillary first molar as previously described^19^. Maxillary tissue and bone overlying the left second molar were removed with an electric drill using a size-4 round bur at a slow speed under saline irrigation. The defect width was standardized to the width of the round bur (1.5 mm in diameter) and extended longitudinally (3 mm) to either side of the exposed root (Fig. 5). The animals were then divided into four groups—a control group and the iPSCs and BMP (10 ng/ml) groups—which each included four animals. A thermosensitive hydrogel mixed with BMP-6 (10 ng/ml) (human recombinant bone morphogenetic protein six, 10 μg, PEPROTECH) was applied to each defect in two experimental groups, and only thermosensitive hydrogel was applied to the control group. After suturing, the wound was left to heal for 42 days. The surgical procedures were standardized, and at the end of experiments, all animals were killed in a 100% CO_2_-filled chamber and the maxillary blocks were obtained for micro-CT and histological examinations.

### Micro-CT assessments

Methods of micro-CT assessments for periodontal defects regeneration were done according to the protocol described by Chang *et al*.^19^. Briefly speaking, the specimens were examined using a Bruker micro-CT System (Skyscan 1076) with an effective pixel size of 8000 × 8000 pixels. After the 3D image reconstruction, the entire osteotomy site was selected semi-automatically as the region of interest (ROI), and the fragments of the root in the ROI were manually excluded by a single-blinded assessor (HCC). The micro-morphometric bone parameters, including bone volume fraction (BVF), trabecular thickness (Tb. Th), and trabecular number (Tb. N), were analyzed by using CTAn software (Skyscan, Kontich, Belgium).

### Histology preparation and histomorphometric analysis

After fixation in 4% paraformaldehyde overnight, the maxillary tissue blocks from four groups (control group, hydrogel-only group, BMP-6 with hydrogel group and iPSCs with BMP-6 and hydrogel group) were subsequently decalcified in 14% ethylenediaminetetraacetic acid, and over the four weeks, the samples were dehydrated in step gradients of ethanol and embedded in methyl methacrylate. Serial horizontal in 5 μm sections represented the entire surgically exposed root surface, and the sections were cut in a buccal-lingual plane throughout the entire mesial-distal extension of the mesial roots of the 1^st^ and 2^nd^ premolars from the coronal to apical aspects that were prepared for histological examination. All the sections were stained with H & E stain (Hematoxylin, Sigma H9627, Eosin, Sigma HT110180). The histometric measurements were performed using a light microscope equipped with a computer-assisted image analysis system (Optimas version 6.2, Media Cybernetics Inc., Silver Spring, MD, USA). The following parameters were recorded and analyzed: defect height, connective tissue repair, new cementum formation, new bone formation and ankyloses.

### Statistical analysis

Statistically significant differences (P < 0.05) between the various groups were measured using ANOVA. All statistical analyses were carried out using the SPSS 11.5 statistical software package (SAS, Cary, NC). All the data were expressed as means with standard deviations.

## Electronic supplementary material

Supplementary information
