# Supplementary material for: Promoting Induced Pluripotent Stem Cell-driven Biomineralization and Periodontal Regeneration in Rats with Maxillary-Molar Defects using Injectable BMP-6 Hydrogel
Source: Sci Rep. 2018 Jan 8;8:114. doi: 10.1038/s41598-017-18415-6 (PMC5758833; doi:10.1038/s41598-017-18415-6)
Supplement: Supplementary file 1 — Supplementary information [file 41598_2017_18415_MOESM1_ESM.doc]

**Promoting Induced Pluripotent Stem Cell-driven Biomineralization and Periodontal Regeneration in Rats with Maxillary-Molar Defects using Injectable BMP-6 Hydrogel**

Ke-Hung Chien^1,2^*, Yuh-Lih Chang^2,3,4*^, Mong-Lien Wang^3,5^, Jen-Hua Chuang^3,5^, Ya-Chi Yang^3,5*^, Ming-Cheng Tai^1^, Chien-Ying Wang^3,5^, Yung-Yang Liu^3,6,7^, Hsin-Yang Li^5,8^, Jiang-Torng Chen^1^, Shou-Yen Kao^9,10^ Hen-Li Chen^9^, Wen-Liang Lo^9,11,12^

^1^Department of Ophthalmology, Tri-Service General Hospital and National Defense Medical Center, Taipei 114, Taiwan;

^2^Institute of Pharmacology, National Yang-Ming University, Taipei 112, Taiwan;

^3^School of Medicine, National Yang-Ming University, Taipei 112, Taiwan;

^4^Department of Pharmacology, Taipei Veterans General Hospital, Taipei 112, Taiwan;

^5^Department of Medical Research, Taipei Veterans General Hospital, Taipei 112, Taiwan;

^6^Institute of Clinical Medicine, National Yang-Ming University, Taipei 112, Taiwan;

^7^Department of Chest, Taipei Veterans General Hospital, Taipei 112, Taiwan;

^8^Institute of Anatomy and Cell Biology, National Yang-Ming University, Taipei 112, Taiwan;

^9^Institute of Oral Biology, National Yang-Ming University, Taipei 112, Taiwan;

^10^Department of Stomatology, Taipei Veterans General Hospital, Taipei 112, Taiwan;

^11^Division of Oral and Maxillofacial Surgery, Department of Stomatology, Taipei Veterans General Hospital, Taipei 112, Taiwan.

^12^Department of Dentistry, School of Dentistry, National Yang-Ming University, Taipei 112, Taiwan.

* Equal Contribution

Corresponding author

**Wen-Liang Lo, DDS, PhD.**

Department of Dentistry, School of Dentistry, National Yang-Ming University, Taipei 112, Taiwan; Division of Oral and Maxillofacial Surgery, Department of Stomatology, Taipei Veterans General Hospital, Taipei 112, Taiwan.

Tel: 886-2-28757572, Fax: 886-2-28742375, E-mail: [wllo@vghtpe.gov.tw](mailto:wllo@vghtpe.gov.tw)

**Supplementary Material and Method**

**Embryoid body preparation from iPSCs**

For preparation of embryoid body (EB) formation, we used 0.25% trypsin-EDTA to make iPSCs dissociated into a single cell suspension and then plated onto non-adherent culture dishes in DMEM with 15% FBS, 100 mM MEM nonessential amino acids, 0.55 mM 2-mercaptoethanol, and antibiotics at a density of 2x10^6^ cells/100 mm per plate. After 4 days in suspension culture, EBs were transferred onto non-adherent Petri dishes and maintained in same medium for another 48 h.

**Real-time Reverse Transcription-polymerase Chain Reaction (RT-PCR)**

Total RNA was extracted with TRIzol Reagent (Thermo Fisher Scientific, Waltham, MA, USA). RNA template (1 μg) was denaturized at 65°C for 5 min, followed by immediate cooling on ice. cDNA was then obtained by reverse transcription using TIANScript RT Kit (Tiangen, Beijing, China) under 37°C for 15 min, followed by enzyme inactivation reaction at 98°C for 5 min. The PCR system (20.0 μl) was composed of 10.0 μl SYBRR Select Master Mix (2X), 0.4 μl forward primer (10 μM), 0.4 μl reverse primer (10 μM), 1.0 μl cDNA template, and 8.2 μl ddH2O.The quantification of unknown samples was performed by LightCycler Relative Quantification Software, version 3.3 (Roche Diagnostics). In each experiment, GAPDH gene was amplified as a reference standard. PCR reactions were prepared in duplicate and heated to 94 °C for 2 minutes, followed by 50 cycles of 94 °C for 20 seconds, 57 °C for 20 seconds, and 70 °C for 20 seconds. All PCR reactions were performed in duplicate. Standard curves (cycle threshold values versus template concentration) were prepared for each target gene and for the endogenous reference (GAPDH) in each sample.

**Immunofluorescence**

For immunofluorescence staining, cells on coverslips were fixed in 4% paraformaldehyde/PBS for 10 to 15 minutes and rinsed with PBS, then permeabilized with 0.1% Triton X-100/PBS for 10 minutes and blocked in 3% BSA for 30 minutes. Coverslips were incubated with primary antibodies for 1 hour at room temperature (to facilitate location in nucleus staining of cells on coverslips, primary antibodies were diluted in 0.2% Triton X-100/PBS and incubated over night at 4°C), washed three times with PBS, and incubated with secondary antibody for 1 hour. The following primary antibodies were used: mouse stage-specific embryonic antigen-1 (SSEA-1; 1:100 dilution; Stemgent), mouse anti-alpha smooth muscle actin antibody (SMA; 1:500 dilution; Abcam), mouse anti-Nestin antibody (Nestin; 1:500 dilution; Abcam), mouse antimicrotubule associated protein 2 (MAP2; 1:300 dilution; Abcam). Coverslips were subsequently incubated with FITC, Alexa Fluor 488, or Cy3-labeled secondary antibodies for 1 hour at room temperature in the dark. After washing three times with PBS for 5 minutes each, samples were counterstained with Hoechst (Invitrogen). Negative controls were stained without primary antibodies. Confocal fluorescence images were acquired with laser scanning microscope (LSM 510; Zeiss, Thornwood, NY).

**MTT assay**

For evaluation of cell survival, cells were seeded on 24-well plates at a density of 2×10^4^ cells/well, followed by the addition of methyl thiazol tetrazolium (MTT; Sigma) at the end of cell culture. The amount of MTT formazan product was determined using a microplate reader at an absorbance of 560 nm (SpectraMax 250, Molecular Devices, Sunnyvale, CA, USA).Statistically signiﬁcant differences (P<0.05) between the various groups were measured using ANOVA. All statistical analyses were carried out using the SPSS 11.5 statistical software package (SAS, Cary, NC). All the data were expressed as means with standard deviations.

**REFERENCES**

[1] Chiou SH, Jiang BH, Yu YL, Chou SJ, Tsai PH, Chang WC, et al. Poly(ADP-ribose) polymerase 1 regulates nuclear reprogramming and promotes iPSC generation without c-Myc. J Exp Med. 2013;210:85-98.

[2] Yang DC, Tsay HJ, Lin SY, Chiou SH, Li MJ, Chang TJ, et al. cAMP/PKA regulates osteogenesis, adipogenesis and ratio of RANKL/OPG mRNA expression in mesenchymal stem cells by suppressing leptin. PLoS One. 2008;3:e1540.

[3] Chiou SH, Kao CL, Peng CH, Chen SJ, Tarng YW, Ku HH, et al. A novel in vitro retinal differentiation model by co-culturing adult human bone marrow stem cells with retinal pigmented epithelium cells. Biochem Biophys Res Commun. 2005;326:578-85.

[4] Chen YC, Hsu HS, Chen YW, Tsai TH, How CK, Wang CY, et al. Oct-4 expression maintained cancer stem-like properties in lung cancer-derived CD133-positive cells. PLoS One. 2008;3:e2637.

**Suppl. Table 1.**

| **The sequences of the primers for quantitative RT-PCR** | | | |
| --- | --- | --- | --- |
| Gene  (Accession No.) | Primer Sequence  (5’ to 3’) | Product  size (bp) | Tm (°C) |
| Sox2  NM_003106.3 | F: ATCAGGAGTTGTCAAGGCAGAG  R: AGAGGCAAACTGGAATCAGGA | 232 | 55 |
| Oct4a  NM_001173531.2 | F: ACATGTGTAAGCTGCGGCC  R: GTTGTGCATAGTCGCTGCTTG | 297 | 55 |
| Nanog  NM_024865.3 | F: TTAATAACCTTGGCTGCCG  R: CCTCCCAATCCCAAACAATA | 150 | 55 |
| Klf4  NM_001314052.1 | F: CACCTTCTTCACCCCTAGA  R: CTCTTTGTGTAGGTTTTGCC | 148 | 55 |
| REX1  NM_174900.4 | F: TTGGGAATTCAGACCTAACC  R: AGTAAGCTGTCTTCAGCAAA | 134 | 55 |
| DPPA2  NM_138815.3 | F: CAATCTCCTTCCATCCCAG  R: CTCATCATCTACTTCCCCCT | 95 | 55 |
| DPPA4  NM_028610.2 | F: CAAAAGAAGTCGGAGACAGA  R: GGAATTGGTACCTTCCTACG | 98 | 55 |
| ESG1  NM_001025290.2 | F: CTCGAATCCCTTACATCGAG  R: CGGAGCTTGTACAAATAGGA | 112 | 55 |
| GDF3  NM_020634.2 | F: CAGACTAAGATGCTCCCTTC  R: TAATGAATAGCTGGTGACGG | 146 | 55 |
| SMAD1  NM_005900.2 | F: CTTTCCAGCAACCCAACAGC  R: GCATCTGGAAAGGGCTTCCT | 132 | 55 |
| SMAD5  NM_001001419.2 | F: ACAGCCCTTATCCCCCTTCT  R: GCTGGGAGCTGAAATGGACT | 88 | 55 |
| ERK  NM_002745.4 | F: AACAGGCCCATCTTTCCAGG  R: CCAGAGCTTTGGAGTCAGCA | 193 | 55 |
| JNK  NM_001278547.1 | F: CTGAAGCAGAAGCTCCACCA  R: CACCTAAAGGAGAGGGCTGC | 159 | 55 |
| P38  NM_001315.2 | F: TACGTGGCCACTAGGTGGTA  R: CAACAGCTCGGCCATTATGC | 108 | 55 |
| GAPDH  NM_002046.5 | F: ACATGTGTAAGCTGCGGCC  R: GTTGTGCATAGTCGCTGCTTG | 83 | 60 |
